# Combining High Oleic Acid Trait and Resistance to Late Leaf Spot and Rust Diseases in Groundnut (*Arachis hypogaea* L.)

**DOI:** 10.3389/fgene.2020.00514

**Published:** 2020-06-10

**Authors:** Dnyaneshwar B. Deshmukh, Balram Marathi, Hari Kishan Sudini, Murali T. Variath, Sunil Chaudhari, Surendra S. Manohar, Ch V. Durga Rani, Manish K. Pandey, Janila Pasupuleti

**Affiliations:** ^1^Professor Jayashankar Telangana State Agricultural University (PJTSAU), Hyderabad, India; ^2^International Crops Research Institute for the Semi-Arid Tropics (ICRISAT), Hyderabad, India

**Keywords:** groundnut, marker-assisted backcrossing, high oleic acid, late leaf spot, rust, disease resistance

## Abstract

High oleic trait, resistance to rust and late leaf spot (LLS) are important breeding objectives in groundnut. Rust and LLS cause significant economic loss, and high oleic trait is an industry preferred trait that enhances economic returns. This study reports marker-assisted selection to introgress high oleic content, resistance to LLS and rust into Kadiri 6 (K 6), a popular cultivar. The alleles for target traits were selected using linked allele-specific, simple sequence repeats and single nucleotide polymorphic markers. The F_1_s (384), intercrossed F_1_s (441), BC_1_F_1_s (380), BC_1_F_2_s (195), and BC_1_F_3_s (343) were genotyped to obtain desired allelic combination. Sixteen plants were identified with homozygous high oleic, LLS and rust resistance alleles in BC_1_F_2_, which were advanced to BC_1_F_3_ and evaluated for disease resistance, yield governing and nutritional quality traits. Phenotyping with Near-Infrared Reflectance Spectroscopy identified three lines (BC_1_F_3_-76, BC_1_F_3_-278, and BC_1_F_3_-296) with >80% oleic acid. The identified lines exhibit high levels of resistance to LLS and rust diseases (score of 3.0–4.0) with preferred pod and kernel features. The selected lines are under yield testing trials in multi-locations for release and commercialization. The lines reported here demonstrated combining high oleic trait with resistance to LLS and rust diseases.

## Introduction

Groundnut or peanut (*Arachis hypogaea* L.) has diversified uses; it is used for food, feed, fodder, and industrial purposes (Sahdev, 2015). It is cultivated across 118 countries covering 27.94 million hectares (Mha) area, with an annual production of 47.09 million tons (MT) of pods. India ranks first with an area of 5.30 Mha, and second in production with 9.17 MT of pods (FAOSTAT, 2017). The demand for groundnut in the food industry is increasing due to its multiple health benefits. The global vegetable oil consumption is expected to double by 2040 including groundnut oil (FAOSTAT, 2015). The groundnut kernel fat consists of monounsaturated fatty acid, oleic acid (36–81.3%), and polyunsaturated fatty acid, linoleic acid (3.9–40.2%) (Janila et al., 2016b). Groundnut kernels with ≥78% oleic acid are considered as high oleic, which contains an oleic to linoleic (O/L) acid fraction of ≥10:1. The consumption of oleic acid-rich diet enhances the ratio of high-density lipoprotein (HDL) to low-density lipoprotein (LDL), thus minimizes the risk of cardiovascular diseases (CVD) by 15% (Wang and Hu, 2017). Palmitic acid, another major fatty acid in groundnut kernels is associated with risk of CVD (Hu et al., 2001). Unlike oleic acid, higher linoleic acid is vulnerable to oxidation causing off-flavors, rancidity, and negatively impacts the oil stability (Patel et al., 2004). Whereas, high oleic groundnut and its products have 5–10 times elevated shelf life than that of normal groundnut, making high oleic groundnut beneficial for oil, food and processing industries.

Spontaneous groundnut mutants (F435-1 and F435-2 with ∼80.0% oleic acid, and 2.0% linoleic acid) were identified at the University of Florida, USA (Norden et al., 1987). Subsequently, the first high oleic line, SunOleic 95R, was bred in the USA (Gorbet and Knauft, 1997). The high oleic trait in groundnut is a result of two homoeologous mutant alleles of *FAD2A* and *FAD2B* on linkage group A09, and B09, respectively, which encode Δ12 fatty acid desaturase (FAD) (Moore and Knauft, 1989; Yu et al., 2008; Chu et al., 2009). In normal groundnut (∼45.0% oleic acid), oleic acid gets converted to linoleic acid by the catalytic activity of FAD. The *FAD2A* mutation involves the substitution of G:C→A:T in the open reading frame (ORF) at 448 base pair (bp) positions on 9th chromosome of A genome, thereby replacing the aspartic acid with asparagine. In the case of *FAD2B* mutation, insertion of adenine at 442 bp in the ORF on 9th chromosome of B genome shifts the ORF resulting in a truncated inactive Δ12 FAD (Yu et al., 2008). These mutations result in the elevated accumulation of high oleic acid as the conversion of oleic to linoleic is stalled and thus decreases linoleic acid. Until now, more than 80 high oleic groundnut varieties are registered globally, which are developed through conventional breeding, marker-assisted selection (MAS), marker-assisted backcrossing (MABC), and mutagenesis (Wang M. L. et al., 2015; Janila et al., 2016b; Bera et al., 2018). In Asian and African market high oleic groundnut has been recently commercialized, however, combining must-have traits such as late leaf spot (LLS), and rust resistance with high oleic are limited (Shasidhar et al., 2020).

Co-occurrence of LLS and rust is common in rainfed ecologies world over, and studies show that in India they together cause 50–70% reduction in pod yield, and reduce the quality and digestibility of haulms up to 22% (Pande et al., 2003; Singh et al., 2011). The fungal pathogens *Phaeoisariopsis personata* (Berk. and Curtis) causes LLS, and *Puccinia arachidis* (Spegazzini) causes rust disease. Although preventative fungicidal spray keeps LLS and rust at bay (Subrahmanyam et al., 1984), fungicides are not feasible in low-input agro-ecologies of Asia and Africa and are not environmentally sustainable. Groundnut crop benefits the farmers through pod yield and adds value through haulms (vines with leaves) as a fodder source for livestock especially in Asia and Africa. In India, groundnut haulms contribute to more than 30% to the fodder obtained from leguminous residues (Nigam and Blummel, 2010). Breeding for disease-resistant varieties is most feasible and environmentally sound approach (Pande et al., 2003).

Host-resistance has been described in cultivated and wild *Arachis* spp. for LLS, and rust (Pande and Rao, 2001; Sudini et al., 2015; Chaudhari et al., 2019). The interspecific derivatives, ICGV 86687 (CS 16–B2–B2), ICGV 86855 (CS 16), ICRISAT (1987) and VG 9514 (Varman, 1999) were developed with high-levels of resistance to LLS and rust. The cultivar GPBD 4 which was bred using CS 16 (interspecific derivative, *A. hypogaea* × *A. cardenasii*) is often used as a donor parent to introgress LLS and rust-resistant quantitative trait loci (QTLs) into popular groundnut varieties. The QTLs governing LLS, and rust resistance were mapped in the recombinant inbred line (RIL) population derived from GPBD 4 (resistant) and TAG 24 (susceptible). The genomic region on chromosome A03 consists of three QTLs for LLS representing about 67.98% phenotypic variation explained (PVE) and rust up to 82.96% PVE (Sujay et al., 2012). The identified QTLs were resolved to 3.06 Mb (131.6–134.6 Mb) on chromosome A03 regions which consisted of ∼25 putative candidate-genes governing LLS and rust resistance (Pandey et al., 2017). The molecular markers for LLS and rust resistance were validated (Sukruth et al., 2015; Yeri and Bhat, 2016) and used to develop LLS and rust-resistant lines using MABC in three susceptible, yet popular varieties *viz.*, TAG 24, ICGV 91114 and JL 24 (Varshney et al., 2014). Six introgression lines with 39–79% higher mean pod yield, and 8–25% higher haulm yield than their recurrent parents were selected based on field evaluation trials (Janila et al., 2016a). More recently three popular varieties from Gujarat, namely GJG 9, GG 20, and GJG-HPS 1 have also been improved for foliar disease resistance in addition to high oleic acid (Shasidhar et al., 2020).

In earlier reports, MABC has been successful in improving groundnuts for nematode resistance (Simpson et al., 2003), rust resistance (Varshney et al., 2014), LLS and rust resistance (Janila et al., 2016a; Yeri and Bhat, 2016; Kolekar et al., 2017; Shasidhar et al., 2020) and enhancing the oleic acid content in groundnut (Janila et al., 2016b; Bera et al., 2018; Shasidhar et al., 2020). Similarly, MABC was deployed to develop lines in groundnut for high oleic content and nematode resistance (Chu et al., 2011); tomato spotted wilt virus (TSWV), peanut root-knot nematode resistance (Holbrook et al., 2017).

Modern crop breeding and improved management practices have enriched 0.8–1.2% of annual gain in crop productivity (Li et al., 2018). In essence, the genetic gain (ΔG) in cultivar improvement program can be elevated by increasing selection intensity (i), additive genetic variation (σ*_*a*_*) existing in the population, selection accuracy (r) and reducing the breeding cycle time (L) (Cobb et al., 2019).

$$\begin{array}{c} \text{Δ}\,G=\left[\left(\sigma_{a}\right)\,\;\,\left(i\right)\,\;\,\left(r\right)\right]/L \end{array}$$

Deploying molecular markers for selection in early generation increases the selection intensity in the breeding population. Rapid generation advancement and resource allocation reduces the breeding cycle time. The selection accuracy can be increased using high-throughput phenotyping tools like Near-Infrared Reflectance Spectroscopy (NIRS) and detached leaf technique.

Kadiri 6 (K 6) is a popular Spanish Bunch groundnut variety has high pod yield, uniform pod size, preferred pod and kernel features, and attractive light tan kernels. K 6 matures in 100–105 days with 70% shelling outturn and 39.0 g sound mature 100-kernel weight. The K 6 kernel contains ∼48.0% oil consist of ∼40.0% oleic acid, and 38.0% linoleic acid. It was developed at Agricultural Research Station, Kadiri, Acharya N. G. Ranga Agricultural University of Andhra Pradesh, India and released for cultivation in the Indian states of Andhra Pradesh, Telangana, and Karnataka in 2005. At present, K 6 covers over 46% Breeder Seed demand in India through public sector seed distribution (AICRP-G, 2018) and is the most popular variety. However, K 6 is highly susceptible to LLS and rust diseases and this study was attempted to combine high oleic trait, LLS and rust resistance in K 6 using the MABC approach.

## Materials and Methods

### Plant Material

A popular Spanish Bunch groundnut cultivar, K 6 was used as recipient parent (RP). The advanced breeding lines- ICGV 15033 [(ICGV 06420 × Sun Oleic 95R) F_2_P402-P1-P1-B1-B1] (Janila et al., 2016b) with an oleic acid content of ca. 82% was used as a donor parent for high oleic trait, while ICGV 13193 {ICGV 91114-P1 × [ICGV 91114-P1 × (ICGV 91114-P1 × GPBD 4-P1_13-1)]} (Varshney et al., 2014; Janila et al., 2016a) with rust and LLS score of 2.0 and 1.0, respectively, at 75 days after sowing (DAS) was used as a donor parent for rust and LLS resistance.

### Hybridization and Generation Advancement

**FIGURE 1:**
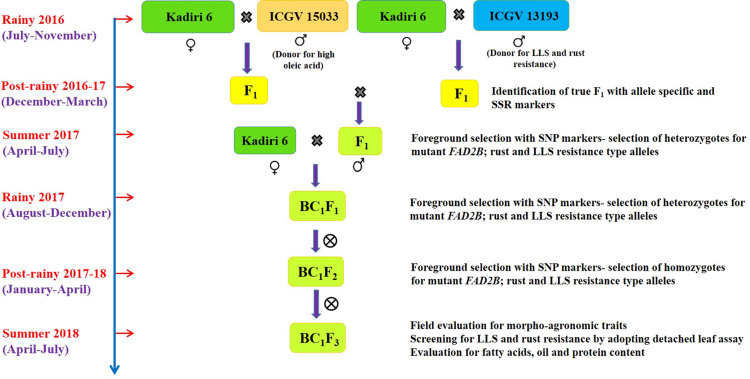
Marker-assisted backcross breeding scheme for improvement of oil quality and resistance to late leaf spot and rust in groundnut *cv*. Kadiri 6.

Two independent crosses were made with K 6 as a pistillate parent and ICGV 15033, and ICGV 13193 as pollen parents (Figure 1). The unopened well-developed basal buds from the female parent were emasculated in the evening (15:00–18:30 h) and pollination was carried out in the next morning (06:00–08:00 h). For pollination, the fully bloomed flower from the male parent was plucked and pollens were gently deposited on the stigma of emasculated flower (Nigam et al., 1990). The population size of F_1_s (384), intercrossed F_1_s (441), BC_1_F_1_s (380), BC_1_F_2_s (195), and BC_1_F_3_s (343) was developed and genotyped to select desired allelic combination.

### DNA Isolation and Genotyping With Linked Markers

The genomic DNA was isolated from the young leaf (50–100 mg) of F_1_s, backcross progenies and parents using modified cetyl trimethyl ammonium bromide (CTAB) method (Mace et al., 2003). DNA was quantified by loading 1 μl on the 0.8% agarose gel containing 10 μl ethidium bromide (10 mg/ml) and run at 80V for 30–45 min. The agarose gel was documented under UV transilluminator. The final DNA quality and quantity were estimated using Nanodrop (Shimadzu UV160A, Japan) and diluted to 5 ng/μl concentration for polymerase chain reaction (PCR). The allele-specific molecular markers positioned on linkage group A09 and B09 were used to select *ahFAD2A* and *ahFAD2B* mutant alleles in F_1_ generation during post-rainy 2016–17 (Table 1 and Figure 1). To track QTL region for disease resistance, earlier reported simple sequence repeat (SSR) markers SEQ8D09 and GMLQ975 were used for LLS, and IPAHM103, GM2301, GMRQ786, and GMRQ843 for rust during post-rainy 2016–17 (Khedikar et al., 2010; Sujay et al., 2012; Pandey et al., 2017).

**TABLE 1:** List of markers used for genotyping of high oleic, late leaf spot, and rust resistance traits.

Trait	Marker name	Linkage group	Sequence (5′-3′)	Allele size (bp)	References
High oleic acid	F435-F (*ahFAD2A*)	A09	Forward: ATCCAAGGCTGCATTCTCAC Reverse (SUB): TGGGACAAACACTTCGTT	M- 203 W- 290	Chen et al., 2010
High oleic acid	F435 (*ahFAD2B*)	B09	Forward: ATCCAAGGCTGCATTCTCAC Reverse (INS): AACACTTCGTCGCGGTCT	M- 190, 290 W- 290	Chen et al., 2010
LLS	GMLQ975	A03	FP: GGTATCATGATGAATTTTTAGAAGACTAGG RP: GAAATTTGGCTTTGGGTTCA	R- 150, 280 S- 280	Sujay et al., 2012
Rust	IPAHM103	A03	FP: GCATTCACCACCATAGTCCA RP: TCCTCTGACTTTCCTCCATCA	R- 154 S- 130	Khedikar et al., 2010
Rust	GM2301	A03	FP: GTAACCACAGCTGGCATGAAC RP: TCTTCAAGAACCCACCAACAC	R- 235 S- 260	Sujay et al., 2012
Rust	GMRQ786 (Gene specific marker)	A03	FP: AACATTGTAACACTCACCTGGCTA RP: TCATGCTTGAACTGTGCCTC	R- 200	Pandey et al., 2017

*FP: Forward primer, RP: Reverse primer, R: resistant, S: susceptible, M: mutant type, W: wild type.*

### Genotyping for LLS and Rust-Resistant Alleles

The touchdown PCR profile was used for screening of LLS and rust-resistant alleles using linked markers. The reaction mixture consisted of 5 ng of DNA, 2 pmol of M13 labeled forward primer, 5 pmol of reverse primer, 2 mM MgCl_2_, 2 mM dNTPs, 0.1 U of Taq DNA polymerase (Kappa Taq) and 1X PCR buffer. A standardized touch down PCR program with 5 min initial denaturation, followed by 5 cycles of 94°C for 20 s, 65°C for 20 s and 72°C for 30 s with 1°C decrement for every cycle, followed by 40 cycles of 94°C for 20 s, constant annealing temperature of 59°C and 72°C for 30 s ending with extension for 20 min at 72°C. The PCR products were resolved on 2% agarose gel for confirmation of amplification. In SSRs, forward primers were dye-labeled with FAM, VIC and NED which were detected as blue, green and black color peaks, respectively, upon capillary electrophoresis. The PCR products were denatured and capillary separated with ABI 3700 automatic DNA sequencer (Applied Biosystems, United States) and GeneMapper Software V (Applied Biosystems) was used to analyze the peak patterns.

### Genotyping for Mutant *ahFAD2* Alleles Controlling High Oleic Trait

The touchdown PCR program described above was used for allele-specific markers. The PCR products were resolved on 2% agarose gel for confirming the amplification. For *ahFAD2A* allele, 203 bp fragment was amplified by F435-F and F435SUB-R in mutant alleles (substitution of G:C to A:T). For *ahFAD2B*, F435-F and F435INS-R amplified 195 bp fragment with insertion mutation (A:T insertion). The primer combination of F435-F and F435-IC-R was used to amplify the 250 bp wild type allele of *ahFAD2A* and *ahFAD2B*.

### Genotyping Using KASP Based SNP Markers

**FIGURE 2:**
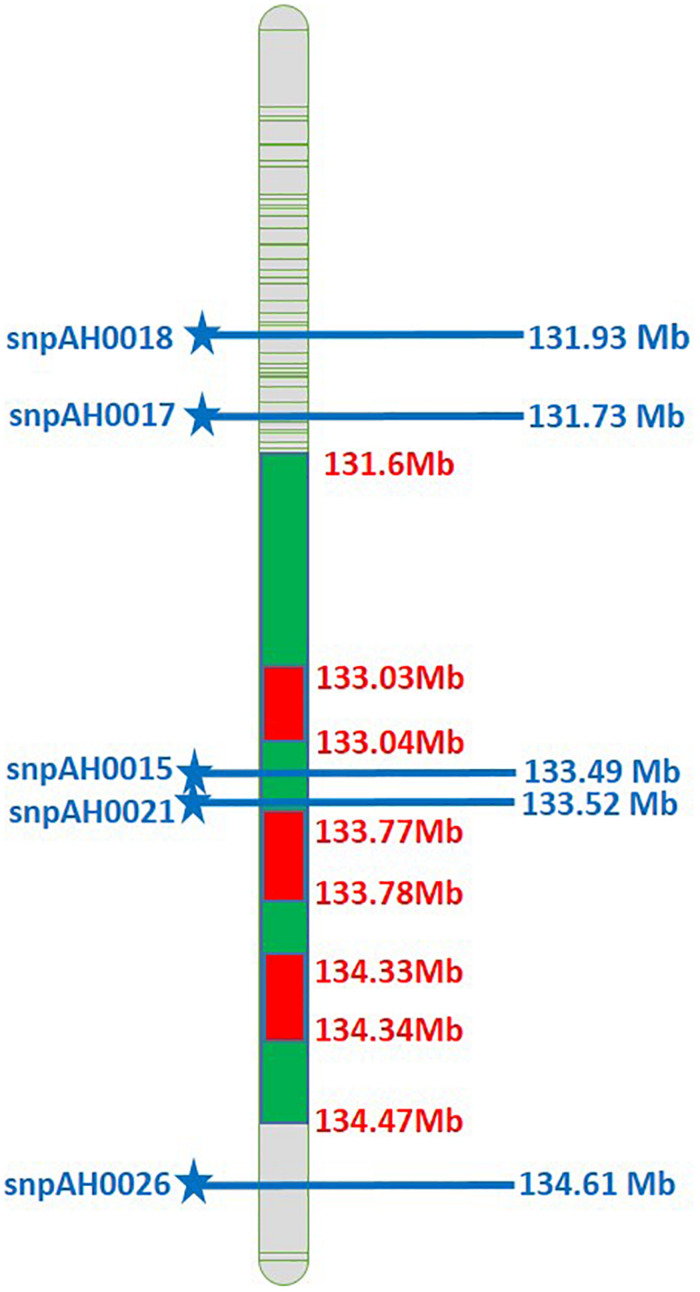
Physical map of A03 comprising the SNPs associated with LLS and rust resistance (Green color represents the QTLs for rust and LLS resistance; Blue color SNPs indicates the physical position of SNPs associated with LLS and rust resistance).

After post-rainy 2016–17, the diagnostic SNPs were utilized for genotyping the breeding population. The SNP markers used in the study consisted one SNP for *ahFAD2B* positioned at +442 bp in the ORF on 9th chromosome of B genome associated with high oleic trait (Pandey et al., unpublished data). The validated five diagnostic SNPs present on A03 (Table 2 and Figure 2) developed by Pandey et al. (2017) associated with LLS and rust resistance were used. The two leaf disks of 5 mm each were collected from each plant for genotyping using Kompetitive allele-specific PCR (KASP^*TM*^) assay.

**TABLE 2:** Selected SNPs associated with late leaf spot, rust resistance and high oleic acid traits used for high throughput genotyping using KASP assay.

SNP ID	SNP code	Trait category	Genomic position (bp)	Target allele (donor type)	Alternate allele
snpAH0002	GKAMFAD2B	Oleic acid content	B sub-genome	A	-:-*
snpAH0015	GKAMA03QR786	LLS and rust resistance	133497786	T	A
snpAH0017	GKAMA03QR517	LLS and rust resistance	131739517	A	C
snpAH0018	GKAMA03QR796	LLS and rust resistance	131937796	G	A
snpAH0021	GKAMA03QR661	LLS and rust resistance	133527661	C	G
snpAH0026	GKAMA03GR173	LLS and rust resistance	134613173	G	C

*-:- Null allele.

### Phenotypic Evaluation for LLS and Rust Resistance Using Detached Leaf Assay

A set of BC_1_F_3’_s along with resistant checks GPBD 4 and ICGV 13193 and susceptible checks Kadiri 6, and TMV 2 were selected for the foliar fungal disease screening using the detached leaf assay (Foster et al., 1980). The fully expanded quadrifoliate third/fourth leaves from the top of a 40-days old plant were excised and washed with deionized water to use as an explant for detached leaf assay. The mixture of sand: vermiculite mixture (1:1 v/v) was prepared and steam-sterilized at 15 lbs pressure and 121°C for 1 hr in two cycles of the autoclave. The experiment was conducted in two replications following a completely randomized design (CRD). The excised explants were implanted in sterile culture (4 cm thick sand) in plastic trays (40 × 30 cm) and sprayed with LLS and rust inoculum (30,000 spores ml^–1^). Transparent polythene sheets were used to cover the trays and incubated in the humidified growth chamber at 24°C with higher relative humidity (∼85%). The leaflets were sprayed once in a day with deionized water and the process was continued up to 8 days after inoculation (DAI). The progress of disease was monitored from 5 to 35 DAI. For LLS resistance, incubation period (IP), latent period (LP), lesion per leaflet (LPL), leaf area damaged in percentage (%LAD), sporulation period (SP) and lesion diameter (LD) were recorded. The parameters *viz*., IP, LP, leaf area damage (LAD), sporulation index and disease scores were recorded to quantify rust resistance. The rust and LLS disease scores were evaluated using modified 1–9 scale at 35 DAI (Subrahmanyam et al., 1995).

### Phenotyping for Morpho-Agronomic and Kernel Quality Traits

The field evaluation of the backcrossed lines was carried out during the post-rainy season of 2018 at red campus West 18 field-block (17.51° North latitude and 78.27° East longitude) under Alfisols (pH 7.0–7.5). Seeds were treated with mancozeb @ 2 g/kg seed and imidachloprid @ 2 ml/kg seed. The air temperature ranged between 22 and 36°C and relative humidity varied between 34 and 87% during post-rainy 2018. The backcrossed lines were planted in 2 m rows. Row to row spacing was maintained at 30 cm while the plant to plant spacing in a row was 10 cm. The basal fertilizer dose of 60 kg phosphorus pent-oxide (P_2_O_5_) was applied. The gypsum was applied @ 400 kg/ha at peak flowering stage of the crop. The lines were harvested at physiological maturity and pods were dried in sunlight for 3 days. The pods were stripped separately from each progeny. The mature-pods from each line were shelled and obtained kernels were used for quality analysis.

The morpho-agronomic traits *viz*., days to 50% flowering, plant height, pod yield per plant, kernel yield per plant, number of pods per plant, shelling outturn, pod length, pod width, pod constriction, pod reticulation, pod beak and pod ridges were recorded by following the groundnut descriptor (IBPGR and ICRISAT, 1992). The oil, protein, and fatty acids were estimated using near-infrared reflectance spectroscopy (NIRS) (XDS monochromator, FOSS Analytical AB, Sweden) for simple, non-destructive, economic and fast screening of progenies. The calibration equation used in prediction had high values of coefficient of determination in external validation (*r*^2^) for oleic acid (*r*^2^ = 0.96), linoleic acid (*r*^2^ = 0.96), and moderate for oil (*r*^2^ = 0.89), protein (*r*^2^ = 0.83) and palmitic acid (*r*^2^ = 0.80) (Murali et al., unpublished data). Seeds obtained from the single plant were scanned twice and data are represented in percentage (%).

## Results

### Marker-Assisted Breeding Scheme

**FIGURE 3:**
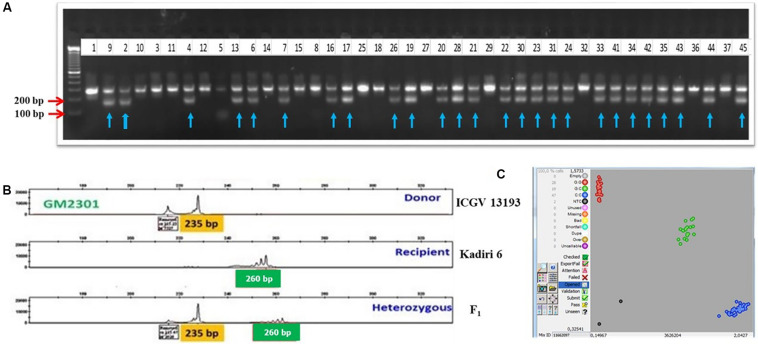
**(A)** Confirmation of zygosity in F_1_ plants derived from a cross between Kadiri 6 and ICGV 13193 using marker GMLQ975 for LLS resistance; Lane 1: Recipient (Kadiri 6), Lane 2: Donor (ICGV 13193), 3–45: F_1_ plants, with arrows indicated for true hybrids. **(B)** Electropherogram showing the resistant type allele (235 bp), susceptible type allele (260 bp) and heterozygous with both the alleles amplified using SSR marker GM2301 for rust resistance. **(C)** Kompetitive Allele Specific PCR (KASP) graphs of SNPs genotyping with snpAH0021 for LLS and rust resistance; (The scatter plot with axes *x* and *y* represents allelic discrimination. Red, green and blue dots represent the susceptible homozygous, heterozygous, and resistant homozygous alleles, respectively).

Two independent crosses were attempted with K 6 as a pistillate parent and ICGV 15033 (oleic acid ca. 82.0%), and ICGV 13193 (LLS and rust-resistant line) as pollen parents to generate F_1_s during rainy 2016. The seeds obtained from the high oleic cross (cross I) were used to raise 210 F_1_s (Table 3) and screened for hybridity using *ahFAD2A* and *ahFAD2B* allele-specific makers. A total of 151 (72.0%) plants showed heterozygous mutant alleles for *ahFAD2A* and *ahFAD2B*. For LLS and rust resistance cross (cross II), 128 F_1_ plants (74.0%) were confirmed with marker GMLQ975 linked to LLS resistance (Figure 3A) and marker GM2301 linked to rust resistance. The amplified PCR product for marker GMLQ975 showed LLS resistant alleles of 150 bp and a susceptible allele of 280 bp. The PCR products amplified using SSR marker GM2301 were separated using capillary electrophoresis followed by electropherogram assay (Figure 3B) which could differentiate recipient and donor type alleles with 260 bp and 235 bp, respectively, and true hybrids showed both the alleles. Positive F_1_s carrying mutant *ahFAD2* alleles, and QTLs for LLS and rust resistance were intercrossed to combine high oleic, LLS and rust resistance. A total of 580 intercrossed F_1_ seeds were obtained of which 441 seeds germinated. The 441 seedlings were genotyped using SNP markers which revealed 44 (10%) true hybrid plants for the high oleic, LLS and rust resistance alleles. Figure 3C represents the differentiation of homozygous resistant (blue dots), heterozygous types (green dots) and homozygous susceptible types (red dots) for snpAH0021 using SNP markers. The positive F_1_ plants (44) were used as a pollen parent to make the backcross with the pistillate parent K 6 and 415 BC_1_F_1_ seeds were harvested.

**TABLE 3:** Summary of hybridization program and marker assisted selection for high oleic trait, LLS and rust resistance in Kadiri 6 derived backcross lines.

Crop season	Cross details	Seedling raised	Confirmed plants for target alleles	Hybridity test output (%)
Post-rainy 2016-17	K6 × ICGV 15033 (Cross I)*	210	151	72
	K6 × ICGV 13193 (Cross II)*	174	128	74
Summer 2017	Cross I × Cross II (Intercross- IC)^†^	441	44	10
Rainy 2017	K 6 × IC-F_1_ (Backcross 1)^†^	380	41	10
Post-rainy 2017–18	BC_1_F${}_{2}^{†}$	195**	16	8

*Plants selected based on gel based markers; ^†^Plants selected based on SNP call.

The hybridity test carried out with SNP markers for 380 BC_1_F_1_s resulted in the identification of 41 positive BC_1_F_1_s, which produced 396 BC_1_F_2_ seeds after self-pollination in rainy 2017. In post-rainy 2017–18, 195 BC_1_F_2_ seedlings were raised and screened for zygosity using SNP markers. The assay revealed 16 plants with homozygous alleles for LLS, rust, and *ahFAD2B* allele which were advanced through selfing to generate BC_1_F_3_ seeds. In post-rainy 2018, these 16 BC_1_F_3_s were raised in the field and evaluated for oil quality, LLS and rust resistance, pod and kernel features. The summary of the data is presented in Table 4.

**TABLE 4:** Summary of Kompetitive allele-specific PCR (KASP) assay for target traits.

SNP combinations	Total genotypes in BC_1_F_2_	Rust score (in BC_1_F_3_)*	LLS score (in BC_1_F_3_)^†^	Oleic acid (%) (in BC_1_F_3_)^¥^
All six target SNPs	16	3–4	3–4	61.2–83.4
High oleic SNP	71	4–7	4–7	60.4–83.4
LLS and rust resistance associated SNPs	45	3–4	3–5	40.6–55.2
Null alleles for LLS and rust resistance and high oleic trait	211	5–8	5–8	38.4–52.6

*-Range of rust score at 35 days after inoculation (DAI), ^†^-range of LLS score at 35 DAI, ^¥^-Range oleic acid content in selected genotypes based on presence of SNPs for target traits.

### Evaluation for LLS and Rust Resistance

**FIGURE 4:**
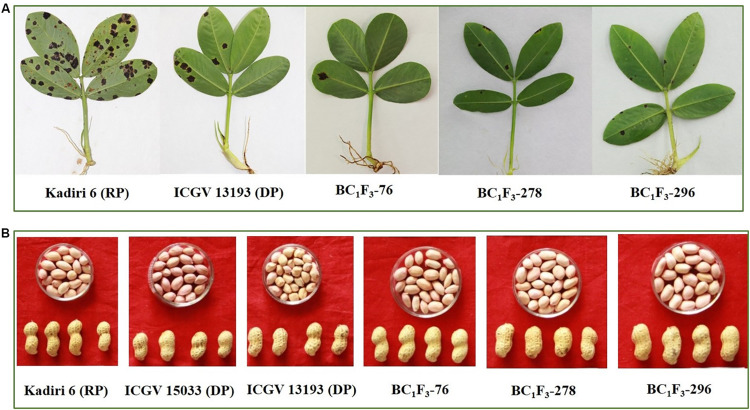
**(A)** Late leaf spot and rust reaction of the recurrent parent (Kadiri 6), donor parent (ICGV 13193) and backcrossed lines at 35 DAI (days after inoculation). **(B)** Pod and kernel features of selected Kadiri 6 derived backcrossed lines.

The results on the screening of fully expanded quadrifoliate leaves using detached leaf assay distinguished resistance, moderate resistance, and susceptibility for LLS and rust (Figure 4A).

#### Late Leaf Spot

The components of LLS resistance were recorded to quantify the resistance of BC_1_F_3_s along with parents and resistant (GBPD 4) and susceptible (TMV 2) checks. The incubation period (IP) varied from 9.5 to 12.8 DAI in backcross lines with highest of 12.8 DAI was recorded in the line, BC_1_F_3_-327, while recurrent parent K 6 recorded 7.0 DAI (Table 5). The sporulation period (SP) varied from 21.5 to 24.7 DAI. The BC_1_F_3_ lines, 144, 186, and 269 showed longer SP of 24.5 to 24.7 DAI as compared to recipient parent, K 6 (15.5 DAI) and susceptible check, TMV 2 (13.5 DAI). The average number of lesions per leaflet (LPL) varied from 19.5 to 78.5 at 35 DAI; the lines BC_1_F_3_-4 (19.5), BC_1_F_3_-5 (27.0), BC_1_F_3_-120 (38.0), and BC_1_F_3_-34 (44.0) recorded a LPS comparable with the donor parent, ICGV 13193 with LPL of 32.5. The recurrent parent recorded an average of 140 LPL. The lesion diameter (LD) in 16 BC_1_F_3_ lines varied from 2.1 to 4.6 mm, while it was 5.7 mm in K 6 and 1.6 mm in ICGV 13193. The lines, BC_1_F_3_-76 (2.1 mm), BC_1_F_3_-296 (2.2 mm), and BC_1_F_3_-225 (2.3 mm) recorded low LD. The percent leaf area damaged by LLS (%LADL) was lower in BC_1_F_3_-296 (15.5%) and BC_1_F_3_-216 (19.5%). The parental lines, K 6 and ICGV 13193 recorded 61.0 and 21.0% LADL, respectively. The disease score of LLS in 16 BC_1_F_3_ lines varied from 3.0 to 5.0. The lines BC_1_F_3_s-76, 216, 225, and 311 were found to be resistant for LLS with a score of ≤3.0 which is at par with donor parent ICGV 13193 (3.0), whereas K 6 recorded a disease score of 8.0 at 35 DAI.

**TABLE 5:** Components of resistance and disease score of Kadiri 6 derived backcross lines (BC_1_F_3_) against late leaf spot (LLS) and rust.

		Late leaf spot						Rust				
S. No.	Genotypes	IP (DAI)	SP (DAI)	LPL (35 DAI)	LD (mm)	% LADL	LLS score (35 DAI)	IP (DAI)	SP (DAI)	PPL (35 DAI)	%LADR	Rust score (35 DAI)
1.	BC_1_F_3_-4	11.5	23.5	19.5	3.3	21.0	4.0	12.5	18.5	23.0	15.5	3.0
2.	BC_1_F_3_- 5	11.5	22.9	27.0	3.5	24.5	4.0	13.5	19.5	21.0	13.0	4.0
3.	BC_1_F_3_- 34	10.0	21.9	44.0	4.5	24.0	4.0	11.5	17.5	21.5	19.0	4.0
4.	BC_1_F_3_-76	10.6	22.7	74.0	2.1	37.7	3.0	11.0	17.0	66.5	24.5	4.0
5.	BC_1_F_3_-120	12.0	23.5	38.0	2.8	24.5	5.0	13.5	19.5	25.0	11.5	4.0
6.	BC_1_F_3_-144	11.5	24.5	72.5	3.5	22.5	4.0	12.5	17.5	22.5	9.5	4.0
7.	BC_1_F_3_-186	9.9	24.5	62.0	2.5	22.7	4.0	13.5	18.0	25.0	21.0	3.0
8.	BC_1_F_3_-214	9.5	22.5	61.5	3.1	24.0	4.0	12.5	16.0	30.5	18.5	4.0
9.	BC_1_F_3_-216	11.5	23.4	76.5	3.4	19.5	3.0	10.0	17.5	26.0	14.5	4.0
10.	BC_1_F_3_-225	11.7	22.7	78.5	2.3	32.5	3.0	12.5	19.5	19.5	12.5	4.0
11.	BC_1_F_3_-249	12.6	23.5	68.6	3.4	21.0	4.0	11.5	19.5	21.5	9.0	4.0
12.	BC_1_F_3_-269	11.6	24.7	63.5	4.6	21.0	4.0	10.5	16.0	37.5	11.0	4.0
13.	BC_1_F_3_-278	9.9	23.5	61.0	2.5	22.5	4.0	12.0	18.0	68.5	20.5	4.0
14.	BC_1_F_3_-296	10.8	21.5	61.5	2.2	15.5	4.0	11.5	15.5	52.0	11.5	4.0
15.	BC_1_F_3_-311	11.9	22.5	55.0	3.1	25.5	3.0	12.5	18.0	29.5	12.5	4.0
16.	BC_1_F_3_-327	12.8	21.5	44.5	4.2	23.0	4.0	12.5	16.5	24.0	13.5	3.0
Checks	TMV 2	7.1	13.5	153.0	6.0	64.5	9.0	8.5	10.5	122.5	40.0	9.0
	GPBD 4	15.6	25.7	13.5	1.2	20.7	3.0	16.5	22.5	12.5	7.5	3.0
DP for LLS & rust	ICGV 13193	11.7	23.5	32.5	1.6	21.0	3.0	14.0	19.0	19.5	8.5	4.0
DP for high O/L	ICGV 15033	9.9	18.5	93.0	4.3	33.5	5.0	12.5	15.5	22.5	10.5	4.0
Recurrent parent	Kadiri 6	7.0	15.5	140.0	5.7	61.0	8.0	9.5	12.0	98.5	25.0	7.0
	SEm ±	1.3	0.4	2.7	0.3	1.2	0.4	0.4	1.1	2.3	1.3	0.4
	LSD (5%)	1.1	1.3	8.0	0.8	3.5	1.2	1.3	3.2	6.8	3.8	1.2

*IP: Incubation Period (days after inoculation), SP: Sporulation Period (days after inoculation), LPL: Lesion Per Leaflet, LD: Lesion Diameter, % LADL: percent Leaf Area Damaged by LLS, PPL: Pustule Per Leaf, %LADR: percent Leaf Area Damaged by Rust, DAI: Days After Inoculation, SEm: Standard Error of the Mean, LSD: Least Significant Difference, DP: Donor parent, LLS: late leaf spot.*

#### Rust

The components of rust resistance were recorded to quantify the level of rust resistance in 16 backcrossed lines. The data of each BC_1_F_3_ lines for various resistance components in comparison with donor and recipient parent are presented in Table 5. The parental lines K 6 and ICGV 13193 recorded IP of 9.5 and 14 DAI, respectively. The IP for rust among BC_1_F_3_s varied from 10.0 to13.5 DAI; long IP of 13.5 DAI was observed in the BC_1_F_3_s-5, 120 and 186. The sporulation period (SP) varied from 15.5 to19.5 DAI; four BC_1_F_3_ lines, 5, 120, 225, and 249 recorded an SP of 19.5 DAI, comparable to the SP of donor parent, ICGV 13193 (19 DAI), whereas the recurrent parent, K 6 recorded an SP of 12.0 DAI. The number of rust pustules per leaflet (PPL) varied from 19.5 to 68.5. Two lines, BC_1_F_3_ 225 (19.5) and BC_1_F_3_-5 (21) recorded the lowest rust PPL comparable with donor parent ICGV 13193 (19.5 PPL), while the RP, K 6 recorded 98.5 rust PPL. The percent leaf area damaged by rust (%LADR) varied from 9.0 to 24.5%; three lines, BC_1_F_3_-249 (9.0%), BC_1_F_3_-144 (9.5%), and BC_1_F_3_-269 (11.0%) recorded low% LADR. The parental lines K 6 and ICGV 13193 recorded a%LADR of 25.0 and 8.5%, respectively. The rust disease score of 3.0 and 4.0 were recorded in BC_1_F_3_ lines, comparable with the score of DP, ICGV 13193 (4.0), whereas the RP, K 6 recorded a score of 7.0 at 35 DAI.

### Evaluation of Backcrossed Lines for Morpho-Agronomic and Kernel Quality Traits

Field screening identified individuals resembling recurrent parent K 6 for morpho-agronomic and kernel features which resulted in the selection of recombinants with at par to superior performance for the selected traits.

### Morpho-Agronomic Traits

The morpho-agronomic performance of 16 BC_1_F_3_ lines of K 6 is presented in Table 6. Days to 50% flowering (DFF) ranged from 31–38 days with an average of 34 days whereas recurrent parent K 6 recorded 35 DFF. The mean plant height was 26.29 cm; it varied from 17.0 cm (BC_1_F_3_-186) to 38.4 cm (BC_1_F_3_-214) and K 6 recorded plant height of 34.6 cm. The mature pods per plant (NPP) varied from 24 to 58; BC_1_F_3_-269 (58) followed by BC_1_F_3_-120 (55) and BC_1_F_3_-144 (54) recorded highest NPP. The weight of mature pods per plant (PYP) varied from 17.2 (BC_1_F_3_-214) to 40.8 g (BC_1_F_3_-269) in comparison with K 6 (26.8 g).

**TABLE 6:** Morpho-agronomic and pod traits of backcross lines of K 6 evaluated during the post-rainy of 2018 at ICRISAT, Patancheru.

S. No.	Genotypes	DFF	PH (cm)	NPP	PYP (g)	SYP (g)	SH (%)	PL (mm)	PW (mm)	PCN	PR	PB	PRG
1.	BC_1_F_3_-4	32.0	29.5	29.0	20.7	14.6	72.0	25.1	10.7	S	S	S	M
2.	BC_1_F_3_-5	32.0	18.5	30.0	28.7	21.5	74.9	25.8	10.6	S	S	S	A
3.	BC_1_F_3_-34	35.0	27.5	30.0	24.0	18.8	78.3	25.9	11.9	M	M	M	S
4.	BC_1_F_3_-76	35.0	21.0	38.0	28.1	20.5	73.0	18.8	8.7	S	S	S	S
5.	BC_1_F_3_-120	34.0	24.5	55.0	33.8	25.1	74.3	19.5	9.3	S	S	S	A
6.	BC_1_F_3_-144	31.0	19.0	54.0	38.0	24.8	65.3	26.4	11.7	S	S	S	S
7.	BC_1_F_3_-186	32.0	17.0	24.0	19.6	15.4	78.6	24.9	10.5	S	S	S	A
8.	BC_1_F_3_-214	34.0	38.4	27.0	17.2	11.8	68.6	27.4	8.9	S	M	S	M
9.	BC_1_F_3_-216	35.0	22.0	38.0	29.1	21.9	74.0	19.8	11.7	S	M	S	S
10.	BC_1_F_3_-225	31.0	29.2	40.0	31.4	24.2	70.0	20.5	10.8	S	S	S	S
11.	BC_1_F_3_-249	32.0	30.4	31.0	25.9	22.0	68.3	21.4	9.9	S	S	S	S
12.	BC_1_F_3_-269	38.0	18.0	58.0	40.8	32.2	78.9	23.0	10.1	S	S	S	A
13.	BC_1_F_3_-278	34.0	29.0	45.0	39.7	29.2	73.6	20.7	9.1	S	S	M	A
14.	BC_1_F_3_-296	36.0	32.5	28.0	21.5	15.7	73.0	25.1	10.5	S	S	S	A
15.	BC_1_F_3_-311	32.0	31.2	42.0	32.2	23.2	72.0	25.5	9.8	S	S	S	A
16.	BC_1_F_3_-327	34.0	33.0	30.0	30.2	20.8	68.9	27.4	8.9	M	S	S	S
17.	ICGV 13193 (DP for LLS & rust)	37.0	29.5	41.0	32.7	28.2	74.0	21.4	11.0	S	S	S	S
18.	ICGV 15033 (DP for high O/L)	36.0	30.0	33.0	25.3	19.3	76.3	20.5	9.8	S	S	S	A
19.	Kadiri 6 (recurrent parent)	35.0	34.6	30.0	26.8	19.8	73.9	24.5	11.5	M	M	P	M
	Mean	34.0	26.2	38.5	29.4	21.3	72.4	23.9	9.9	–	–	–	–
	Range	31.0–38.0	17.0–38.4	24.0–58.0	17.2–40.8	11.8–32.2	65.3–78.9	18.8–27.4	8.7–11.9	–	–	–	–

*DFF: Days to 50% Flowering, PH: Plant Height (cm), NPP: Number of Pods per Plant, PYP: Pod Yield per Plant (g), SYP: Seed Yield per Plant (g), SH (%): Shelling outturn (%), PL: Pod Length (mm); PW: Pod Width (mm), PCN: Pod Constriction, PR: Pod Reticulation, PB: Pod Beak, PRG: Pod Ridges, S: Slight, M: Medium, P: Prominent, A: Absent, DP: donor parent, LLS: late leaf spot.*

The mean seed weight per plant (SYP) was 21.3 g with a range of 11.8–32.2 g. The highest SYP was recorded in BC_1_F_3_-269 (32.2 g) as compared to K 6 (19.8 g). Shelling out-turn (SH) varied from 65.3 to 78.9% with a mean of 72.4%. The higher SH was recorded in BC_1_F_3_-269 (78.9%) followed by BC_1_F_3_-186 (78.6%) and BC_1_F_3_-34 (78.3%). The length of mature pods (PL) in BC_1_F_3_s-214 and 327 (27.4 mm) was comparable to K 6 (24.5 mm). The width of mature pods (PW) was measured 8.7 mm (BC_1_F_3_-76) to 11.9 mm (BC_1_F_3_-34). Recurrent parent K 6 recorded an average PW of 11.5 mm. The recurrent parent had pods with moderate constriction. In the study, a total of 14 lines were observed with slight pod constriction whereas two lines (BC_1_F_3_-34 and BC_1_F_3_-327) were observed with moderate constriction similar to K 6. Presence of slight constriction is preferred over moderate and/or prominent constriction, hence the selection of pods with lesser constriction is preferred to further improve K 6 for preferred pod features along with target traits. Pod reticulation was slight in the selected lines. Pod beak was slight in two lines (BC_1_F_3_s-76 and 296) and medium in BC_1_F_3_-278. Similarly, pod ridges were absent in BC_1_F_3_s- 278 and 296 and, whereas slight in BC_1_F_3_-76.

### Kernel Quality Traits

#### Oil Content (%)

The kernel oil content in back cross lines varied from 46.0 to 54.9% and the recurrent parent, K 6 recorded an oil content of 49.5%. The higher oil content (>50.0%) was observed in 11 lines (BC_1_F_3_s-5, 76, 120, 144, 186, 214, 225, 249, 278, 296, 327).

#### Fatty Acid Content (%)

The oleic, linoleic, and palmitic acid were estimated using NIRS. The oleic acid content varied from 62.5 to 83.4% and the linoleic acid varied from 25.4 to 43.5%. The oleic acid content of >80% was observed in three lines BC_1_F_3_-76 (81.3%), BC_1_F_3_-278 (82.8%) and BC_1_F_3_-296 (83.4%). The recurrent parent, K6 recorded an oleic acid content of 39.8%. The selected lines recorded lower linoleic acid of 9.3% compared to the recurrent parent, K 6 (37.4%). The oleic: linoleic acid (O/L) ratio was 5.2 to 17.1; the ratio in the selected lines, BC_1_F_3_-76, 278 and 296 is >10.

The palmitic acid varied from 7.5 to 11.1% with an average of 9.6% indicating a reduction to an extent of 2.2–5.8% compared to the recurrent parent, K 6 (13.3%). The high oil content with elevated oleic acid and reduced linoleic and palmitic acid is desirable for the improvement in oil quality of groundnut. The present study identified three lines BC_1_F_3_s-76, 278 and 296 with high oleic acid (≥80.0%) and high oil (>50.0%) content indicating that these segregants can be used to develop a high oil and oleic variant of K 6.

#### Protein Content (%)

The protein content of the BC_1_F_3_ lines varied between 23.3 and 29.5%; the protein content of selected lines is BC_1_F_3_-278 (29.5%), BC_1_F_3_-216 (28.9%) and BC_1_F_3_-327 (28.1%), while that of RP, K 6 is 26.6%.

### Superior Segregants for Late Leaf Spot and Rust Resistance, Agronomic, and Kernel Quality Traits

**FIGURE 5:**
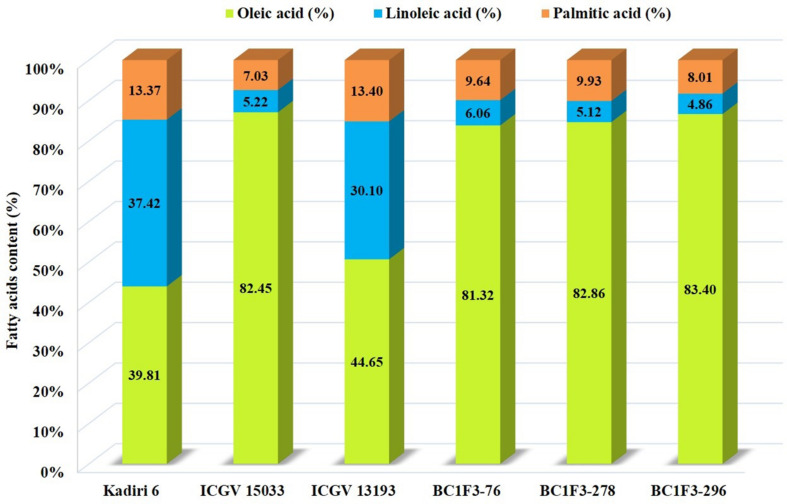
Fatty acid profiles of parental lines and selected backcrossed lines.

**FIGURE 6:**
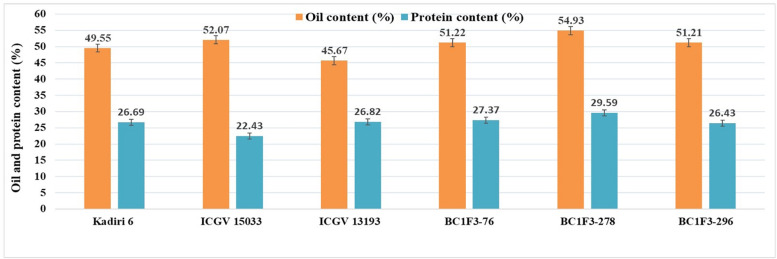
Oil and protein contents of selected Kadiri 6 derived backcrossed lines.

The combined approach MABC and phenotypic-based screening in BC_1_F_3_s was found to be effective in developing and selecting superior segregants for LLS and rust resistance, desired pod features, and improved oil quality traits. The lines BC_1_F_3_-76, BC_1_F_3_-278 and BC_1_F_3_-296 were found at par to marginally superior to the recurrent parent (Table 7 and Figure 4B). The LLS and rust score of the selected lines at 35 DAI varied from 3.0 to 4.0, and 4.0, respectively, indicating good level of resistance. The day to 50% flowering in lines was 35 days in BC_1_F_3_-76, 34 days in BC_1_F_3_-278 and 36 days in BC_1_F_3_-296 comparable to the recurrent parent, K 6 (35 days). The number of pods per plant was 38 and 45 for BC_1_F_3_-76 and BC_1_F_3_-278, respectively, as against 30 for K 6. The selected lines had pod yields ranging from 21.5 to 39.7 g per plant, while it was 26.8 g for K 6. The pod characteristics including pod constriction and pod reticulation were slight in all three selected backcross lines, pod beak was slight in BC_1_F_3_s-76 and 296 and medium in BC_1_F_3_-278, pod ridges were absent in BC_1_F_3_s-278 and 296 whereas slightly present in BC_1_F_3_-76. The oleic acid content in the selected lines was high and ranged from 81.3 to 83.4% (Figure 5). All the selected lines showed higher oil content (>50.0%), protein content (>26.0%) (Figure 6), oleic acid (>80.0%) and reduced linoleic acid content (<10.0%).

**TABLE 7:** Late leaf spot and rust reaction, agronomic performance, and pod characteristics of selected superior lines.

Genotypes	Disease score (35 DAI)		Agronomic traits and oleic acid content							Pod characteristics					
	LLS score	Rust score	DFF	PH	NPP	PYP	SYP	SH	OA	PL	PW	PCN	PR	PB	PRG
BC_1_F_3_-76	3.0	4.0	35.0	21.0	38.0	28.1	20.5	73.0	81.3	18.8	8.7	S	S	S	S
BC_1_F_3_-278	4.0	4.0	34.0	29.0	45.0	39.7	29.2	73.6	82.8	20.7	9.1	S	S	M	A
BC_1_F_3_-296	4.0	4.0	36.0	32.5	28.0	21.5	15.7	73.0	83.4	25.1	10.5	S	S	S	A
ICGV 13193	3.0	4.0	37.0	29.5	41.0	32.7	28.2	74.0	44.6	21.4	11.0	S	S	S	S
ICGV 15033	5.0	4.0	36.0	30.0	33.0	25.3	19.3	76.2	82.4	20.5	9.8	S	S	S	A
Kadiri 6	8.0	7.0	35.0	34.6	30.0	26.8	19.8	73.8	39.8	24.5	11.5	M	M	P	M

*DAI: Days After Inoculation, LLS: Late Leaf Spot, DFF: Days to 50% Flowering, PH: Plant Height (cm), NPP: Number of Pods Per Plant, PYP: Pod Yield per Plant (g), SYP: Seed Yield per Plant (g), SH: Shelling Outturn (%), OA (%): Oleic acid content, PL: Pod Length (mm); PW: Pod Width (mm), PCN: Pod Constriction, PR: Pod Reticulation, PB: Pod Beak, PRG: Pod Ridges, S: Slight, M: Medium, P: Prominent, A: Absent.*

## Discussion

To meet the market needs of groundnut, kernel quality is an important trait and combining it with productivity traits such as biotic and abiotic stress tolerance is an important objective of groundnut breeding program. The high oleic trait is preferred by the food industry for increased shelf-life and consumer health benefits. The co-occurrence of *P. personata* and *P. arachidis* pathogens causes necrotic lesions, defoliation, and rust pustules, respectively, reducing the pod yield by 50–70%, and indirectly affecting kernel and haulm quality.

In the present study, the allele-specific markers were utilized to distinguish mutant and wild-type alleles of *ahFAD2A* and *ahFAD2B* for the high oleic trait (Chen et al., 2010). These markers were successfully deployed in groundnut breeding programs in the USA, Argentina, China and India to develop high oleic groundnut varieties (Chu et al., 2011; Mienie and Pretorius, 2013; Xiuzhen et al., 2016; Janila et al., 2016b; Bera et al., 2018; Shasidhar et al., 2020). For LLS and rust resistance, SSR markers (Sujay et al., 2012) were utilized during the first season of the crossing program. The KASP assay facilitates the plant breeding program with the use of a few SNPs to screen many samples and has an advantage of cost, simplicity, and speed (Chopra et al., 2018). The KASP assay has been successfully deployed in groundnut for rust resistance (Leal-Bertioli et al., 2015), nematode resistance (Chu et al., 2016), high oleic trait (Chopra et al., 2015; Zhao et al., 2017), bacterial wilt resistance (Zhao et al., 2016), LLS resistance (Clevenger et al., 2018), plant architectural traits (Chopra et al., 2018), early leaf spot and LLS, and TSWV (Agarwal et al., 2018) and for LLS, rust and high oleic traits at ICRISAT (Murali et al., 2019).

The gene introgression using MAS/MABC has been deployed in many crops to improve an elite cultivar through introgression of target traits from one or many donors (Tyagi et al., 2014). The parallel crossing of a recurrent parent with donor parents and intercrossing F_1_s followed by backcross with a recurrent parent is a promising way of combine multiple traits. This scheme was utilized in combining traits like bacterial leaf blight (BLB) and lepidopteran insect pest tolerance in rice (Jiang et al., 2004), BLB in rice (Shanti et al., 2010), wheat stripe rust and leaf rust resistance genes (Santra et al., 2006; Yadawad et al., 2017), barley leaf rust, net blotch and spot blotch resistance (Hickey et al., 2017).

This is the most reliable method as it reduces the time period to combine genes/QTLs and assures the fixation of genes/QTLs (Joshi and Nayak, 2010). The normal breeding of an elite cultivar typically takes 6–8 breeding cycles and another 3–5 years of testing for release or commercialization. The use of molecular markers, and improved high-throughput phenotyping methods help the breeders to develop new cultivars in a shorter time span thereby enhancing the rate of genetic gain. We successfully introgressed high oleic, and resistance to LLS and rust trait into popular cultivar K 6 within a 2-year period using high-throughput genotyping and phenotyping coupled with rapid generation advancement. In this study, the breeding cycles per year has been increased from two to three. This resulted in a reduction in breeding cycle time (L) from 0.5 (two cycles per year) to 0.33 (3 cycles per year), thus significantly enhancing the rate of genetic gain.

In MABC, up to four backcrosses are suggested to retain the yield and quality features of the recurrent parent (Sundaram et al., 2008). Moreover, extra cycles of backcrossing have leverage when the adaptation of donor genotypes (wild relatives/germplasm/landraces) is poor. In the present study, the donor parents, ICGVs 15033 and 13193 possess good agronomic characters with a higher yield potential (Janila et al., 2016a, b). Even though repeated cycles of backcrossing can increase the recurrent parent genome recovery, each additional backcrossing could result in the reduction of minor QTLs/genes that leads to moderate expression of target traits in introgressed lines (Wang et al., 2009). Kolekar et al. (2017) observed more recovery of resistant progenies for rust over the LLS, especially when the target trait is controlled by a combination of large and small effect QTLs. A strategy of single backcrossing has been successfully used to introgress rust-resistant genes in wheat (Wang et al., 2009; Yadawad et al., 2017), leaf rust, net blotch, and spot blotch resistance in barley (Hickey et al., 2017), and LLS and rust resistance in groundnut (Kolekar et al., 2017). These studies indicated that one to two cycles of backcross is sufficient in improving elite cultivars while transferring the desired genes from an adapted donor parent. A single backcross can recover an average of 75% of the recipient genome.

The KASP genotyping platform enabled the early selection of plants for crossing thus facilitated generation advancement. The application of MAS in early cycles of breeding helps to detect the favorable introgressions from the donor parents and helps to reduce the population size (Collard and Mackill, 2007). We generated 380 BC_1_F_1_ plants from which 41 were selected based on presence all the three alleles. Even though the population of 195 BC_1_F_2_ plants was low to generate desirable segregants, hence we had a backup program where we have advanced heterozygotes too. Nonetheless, we could identify promising lines from the limited F_2_ population which can be attributed to the use of two elite lines as parents in the crossing.

Detached leaf assay is a rapid and simple method used to screen groundnut genotypes for LLS resistance (Foster et al., 1980), and offers an advantage of off-season screening. In the present study, detached leaf assay was used to evaluate BC_1_F_3_s for LLS and rust resistance under controlled conditions. The detached leaves were placed in a culture of sand: vermiculite (1:1v/v) which has the advantages of maintenance of optimum moisture and relative humidity (≥85%), with minimum contamination of saprophytes and secondary infection. Other commonly used methods include detached leaf culture in Hoagland’s solution (Foster et al., 1980), whole seedling assay planted in pots (Dwivedi et al., 2002) and sand culture (Janila et al., 2013). For artificial inoculations, the spore concentration of ∼30,000 conidia ml^–1^ was maintained. Motagi (2001) suggested the spore suspension of ≥20,000 conidia ml^–1^ is sufficient to build the proper inoculum load to screen groundnut entries for LLS, and rust resistance. Dwivedi et al. (2002) reported that the disease reaction of detached leaflets inoculated with the artificial inoculum determined using disease incidence and severity values were similar to the reaction of the intact plant. This indicates the reliability of the resistant lines identified in the study.

The phenotyping for pod and kernel characteristics including kernel quality helps to select the progenies with a desirable combination of traits as most of these features are monogenically inherited (Bera and Das, 1998; Reddy et al., 2017). Pod and kernel features in groundnut are important for overall appearance and for fetching higher market value, hence considered as selection criteria along with higher pod yield. In the present study, three lines (BC_1_F_3_s-76, 278 and 296) were obtained with superior pod shape over the recurrent parent. Fourteen backcross lines featured with slight beak whereas two lines (BC_1_F_3_s-34 and 278) showed moderate beak. Selection of pods without beaks is an important feature as pods with beaks are susceptible to cracking in soil paving the way for the entry of *Aspergillus* group of fungi and subsequent aflatoxin contamination. After drying, pods sometimes crack toward the beak region making the kernels vulnerable for entry of storage pest and molds (Okello et al., 2010). The recipient parent K 6 had a prominent beak (Figure 4B), an undesirable feature that can be a potential source for the entry pathogens such as, *Aspergillus* Sp., hence priority was given to select the lines with pods with the slight beak.

The kernel oil, protein, and fatty acid content are important criteria in trade, food, and seed industries of groundnut. The wet chemical methods for the analysis of oil, protein and fatty acids content are accurate but operationally complex, time-consuming, destructive and expensive (Sundaram et al., 2009). The relative proportion of three fatty acids namely, palmitic, oleic, and linoleic acid determine the kernel quality and shelf-life. Janila et al. (2016b) and Bera et al. (2018) observed that the introgression of mutant *ahFAD2* alleles into elite breeding lines leads to increase in oleic acid (≥80.0%) and reduced accumulation of linoleic acid (≤8.0%) and palmitic acid (≤10.0%). The palmitic acid (C16:0), a saturated fatty acid contributes to ca. 10.0% of the kernel fat, and the remaining is shared by other minor fatty acids (Wang X. Z. et al., 2015). Normal groundnut oil (∼45% linoleic acid) is vulnerable to oxidation if stored for longer period, resulting in unpleasant smell, and taste of the oil and groundnut products. Hence, groundnut oil and its products with a high O/L are preferable (Vassiliou et al., 2009).

The high oleic acid content of ca. 80% is conferred by a combination of two mutant alleles, *ahFAD2A* and *ahFAD2b*. In the present study, the BC_1_F_3_ lines were selected based on the homozygosity for *ahFAD2B* mutant allele, hence the selected lines recorded an oleic acid content variation of 61.2–83.4%. Thus, the lines that carried *ahFAD2A* mutant allele along with *ahFAD2B* recorded high oleic acid content of ca. 80%. Selection for *ahFAD2A* allele was done by phenotype using NIRS in the present study. NIRS is a non-destructive technique of estimating fatty acids, which includes the standard error in prediction (of up to 5%), due to the lesser quantity and poor quality of seeds used in estimation. Insufficient quantity and poor quality of seeds in early generations can reduce the prediction accuracies, hence confirmation in the later generation when the seed quantity is large is desirable. The accurate estimations are necessary for the commercialization purpose and it is recommended to use gas chromatography (GC). The selected lines with >80% high oleic acid content recorded a variation to an extent of 2% and similar results were also reported by Janila et al. (2016b). Hamdan et al. (2009) reported the loss of minor effect QTLs or modifying genes resulted in variation in the oleic acid content even toward the higher side. The variation in oleic content could also relate to incomplete dysfunctional mutant *ahFAD2B* allele. There could be possible involvement of other unidentified genetic factors or enhancers conditioning oleic acid content in groundnut (Wang X. Z. et al., 2015).

The benefit of phenotypic screening coupled with MAS in early generations facilitates selection for a trait/s (Hickey et al., 2017). Even though, SNP markers confirmed the presence of LLS and rust governing major effect QTLs, information on genes with minor effects and genetic modifiers necessary to achieve higher levels of LLS resistance is limited. The LLS resistance is a genetically complex mechanism, governed by a combination of component traits such as reduced sporulation, latent period, and smaller lesion diameters (Dwivedi et al., 2002). The rust resistance in groundnut was reported due to the accumulation of phytoalexins and elevated proteinases 1,3-α-glucanases, and 1,3-β-glucanases (Sankara et al., 1996). The insights on physiological and biochemical components of resistance to LLS and rust and yield trade-off, if any in resistant cultivars can be future avenues of research. Broadening the genetic base for LLS and rust resistance can provide the broad-spectrum resistance against LLS and rust. This can contribute to improved selection for LLS and rust resistance and modeling growth and yield responses of groundnut.

The backcross-derived populations bred in the present study showed improved resistance to LLS and rust. These lines were higher or at par for yield governing traits in comparison to K 6. Adoption of more efficient selection strategies such as high throughput genotyping, NIRS for oil quality traits is critical for selection in early generations. This approach could be useful for the rapid transfer of disease resistance and high oleic trait into popular groundnut cultivars grown by farmers.

Development and release of high oil and high oleic groundnut varieties have tremendous market potential both in India as well as globally. The promising breeding lines identified in the study will be advanced and evaluated in replicated field trials for disease resistance, high oleic acid and yield traits. Advanced superior breeding lines derived from K 6 for LLS, rust resistance and high oleic traits will be a valuable resource either for use in crossing program or for release as a variety. The above results demonstrated the effectiveness of the molecular marker for early generation selection, and to enhance selection intensity as the number of selected candidates for the same number of selected individuals is higher when we used markers. Selection intensity is directly proportional to genetic gain and thus the use of MABC resulted in an increase in genetic gain. The process also demonstrated enhanced selection accuracy (r) by using high throughput phenotyping, such as NIRS for assessing quality parameters and detached leaf technique for studying disease reaction.

## Data Availability Statement

The genotype data is uploaded in institutional repository with the DOI number: https://doi.org/10.21421/D2/FWIWWC.

## Author Contributions

DD conducted the experiment and drafted the manuscript. BM, CR, and JP conceived and designed the experiment. HS helped with disease screening and manuscript editing. MP performed foreground selection using linked markers for oleic acid, and rust, and LLS, and helped in the manuscript editing. JP, MV, SC, and SM helped with the experiment and critical review of the manuscript.

## Conflict of Interest

The authors declare that the research was conducted in the absence of any commercial or financial relationships that could be construed as a potential conflict of interest.
